# Association of the Estrogen Receptor 1 Polymorphisms rs2046210 and rs9383590 with the Risk, Age at Onset and Prognosis of Breast Cancer

**DOI:** 10.3390/cells12040515

**Published:** 2023-02-04

**Authors:** Heidi Miedl, Denise Oswald, Isabella Haslinger, Manuela Gstoettner, René Wenzl, Katharina Proestling, Christian Schneeberger, Iveta Yotova, Martin Schreiber

**Affiliations:** 1 Department of Obstetrics and Gynaecology, Medical University of Vienna, 1090 Vienna, Austria; 2 Comprehensive Cancer Center (CCC), Medical University of Vienna, 1090 Vienna, Austria

**Keywords:** breast cancer, estrogen receptor, *ESR1*, ERα, single nucleotide variant (SNV), single nucleotide polymorphism (SNP), rs2046210, rs9383590, survival, age at onset

## Abstract

Estrogen receptor α (ERα), encoded by the *ESR1* gene, is a key prognostic and predictive biomarker firmly established in routine diagnostics and as a therapeutic target of breast cancer, and it has a central function in breast cancer biology. Genetic variants at 6q25.1, containing the *ESR1* gene, were found to be associated with breast cancer susceptibility. The rs2046210 and rs9383590 single nucleotide variants (SNVs) are located in the same putative enhancer region upstream of *ESR1* and were separately identified as candidate causal variants responsible for these associations. Here, both SNVs were genotyped in a hospital-based case-control study of 409 female breast cancer patients and 422 female controls of a Central European (Austrian) study population. We analyzed the association of both SNVs with the risk, age at onset, clinically and molecularly relevant characteristics and prognosis of breast cancer. We also assessed the concordances between both SNVs and the associations of each SNV conditional on the other SNV. The minor alleles of both SNVs were found to be non-significantly associated with an increased breast cancer risk. Significant associations were found in specific subpopulations, particularly in patients with an age younger than 55 years. The minor homozygotes of rs2046210 and the minor homozygotes plus heterozygotes of rs9383590 exhibited a several-years-younger age at onset than the common homozygotes, which was more pronounced in ER-positive and luminal patients. Importantly, the observed associations of each SNV were not consistently nullified upon correction for the other SNV nor upon analyses in common homozygotes for the other SNV. We conclude that both SNVs remain independent candidate causal variants.

## 1. Introduction

Genome-wide association studies (GWAS) have demonstrated that single nucleotide variants (SNVs) in chromosomal region 6q25.1, which contains the *ESR1* gene, are significantly associated with breast cancer susceptibility [1,2]. rs2046210 (with genotypes GG, GA and AA) has been referred to as “Asian lead SNV”, since its association with breast cancer risk was initially discovered in a Chinese study population [1]. Subsequently, the minor allele (A) of rs2046210 was confirmed to be associated with an increased breast cancer risk in women of European ancestry as well [1,2,3]. An analysis of the association of rs9383590 (with genotypes TT, TC and CC) with the risk of breast or other cancers has not been reported thus far.

Fine-scale mapping and comprehensive association analysis in a 1 Mb genomic region containing the *ESR1* gene revealed five regions with a size of ≈3–20 kb (termed signals) upstream, within introns or downstream of the *ESR1* gene, in which genetic variants with a strong association with breast cancer susceptibility were highly enriched [4]. This study also provided evidence for the existence of at least one and possibly more than one independent causal variant in each of these five regions [4]. Accordingly, the strength of association of the most significantly associated SNV of region 1 was only somewhat reduced, but it was not nullified upon correction for four other lead SNVs (one each, located in regions 2–5), and the same was observed for the SNVs of all five regions [4]. The SNVs in regions 1, 2, 4 and 5 were independently associated with the risk of estrogen receptor (ER)-negative breast cancer, and the SNVs in regions 1, 2, 3 and 5 were associated with the risk of ER-positive breast cancer [4]. rs2046210 is located 29.4 kb upstream and rs9383590 is located 24.0 kb upstream of the first exon of *ESR1* in region 1, the 5′ most of those five regions relative to the *ESR1* gene [1,4,5].

Functional analyses aimed at a mechanistic explanation for the association of SNVs at 6q25.1 with breast cancer risk were also reported, focusing on their potential role in the regulation of the expression of *ESR1* and possibly additional nearby genes such as CCDC170 [4]. Here, we summarize the results obtained for rs2046210, rs9383590 and region 1. The risk allele of rs2046210 (A) was found to be associated with significantly reduced ERα protein levels in normal breast tissue and in normal tissue adjacent to ER-negative, but not ER-positive, tumors. Furthermore, allelic imbalances in *ESR1* mRNA expression were found in rs2046210 heterozygotes [4,6]. In contrast, no association of rs9383590 with *ESR1* expression was found [7]. In silico and in vitro analyses revealed that elements within region 1, including an element containing rs9383590, overlapped with DNase I hypersensitive sites and enhancer-enriched histone marks and engaged in long-range physical interactions with the promoters of *ESR1* and additional adjacent genes [4,7]. These findings indicate that region 1 contains enhancer element(s) involved in the regulation of *ESR1* expression, which was confirmed in reporter assays. These reporter assays also showed reduced enhancer activity for the minor (risk) alleles of two SNVs in region 1 (not including rs2046210 or rs9383590) [4]. rs9383590 is located in a binding site for transcription factor GATA3, and allele-specific chromatin immunoprecipitation (ChIP) showed a reduced binding of GATA3 to the minor allele (C) of rs9383590 [7]. Consistently, the CRISPR-Cas9-mediated deletion of a 2.2 kb fragment which contained a 1 kb part of region 1 including rs9383590 resulted in a significant decrease in *ESR1* expression, indicating a direct causal role of this region and possibly rs9383590 in *ESR1* expression [7]. Accordingly, rs9383590 has been suggested to be a functional SNV in the *ESR1* locus that accounts for its association with breast cancer risk [7].

However, association analyses of rs9383590 itself are still lacking, as is a systematic comparison of both SNVs in the same study population and with the same methodology. Accordingly, the major objective of this study was a comprehensive analysis of the association of rs2046210 and rs9383590 with the risk, age at onset, clinically and molecularly relevant characteristics and prognosis of breast cancer. In particular, the concordances and discordances between both SNVs, as well as the associations of each SNV conditional on the other one, were investigated. These two SNVs were selected for our study because (i) they are located in the same enhancer region; (ii) strong evidence was reported indicating that rs9383590 is the functional SNV of this region; (iii) rs2046210 was most significantly associated with the risk of ER-negative breast cancer among 3872 SNVs in and around the ESR1 locus that were analyzed in a large epidemiological study [4,7]. Importantly, rs2046210 and rs9383590 were thus identified as potential causal variants in two independent studies with different study populations and methodologies, calling for a side-by-side comparison reported here [4,7]. Our pre-specified hypothesis based on these reports was that rs9383590 is the causal SNV of this region, and the associations reported for rs2046210 are indirect effects of its close proximity and strong linkage disequilibrium with rs9383590.

## 2. Materials and Methods

### 2.1. Study Population

Only women of Central European descent from the same geographical area were included in this study. Healthy females and consecutive patients with benign gynecological lesions and without any malignancies (breast or other cancer) in their personal history were enrolled as nested controls between 2002 and 2004 at the Department of Obstetrics and Gynecology, MUV (*n* = 255). Another 169 controls were enrolled between 2013 and 2017 at the Department of Obstetrics and Gynecology (MUV) in the EMMA study of endometriosis [8]. The EMMA study comprises 280 control subjects, but 20 were excluded due to the lack of a blood sample suitable for DNA isolation, 21 due to non-European descent and 70 due to an age under 30 years. A total of 276 consecutive female breast cancer patients treated between 2002 and 2004, and another 134 consecutive patients treated between 1989 and 1993 at the Department of Obstetrics and Gynecology, MUV, were enrolled in this study. From the latter 134 patients, detailed follow-up records were available (the end of the follow-up period was September 2005). The patient characteristics, follow-up details and adjuvant therapies of these 134 patients have been described previously [9]. The malignant breast cancer of all patients was confirmed by histopathology. None of the patients received any neoadjuvant or other therapy prior to tumor tissue isolation. Tables S1 and S2 depict the clinical and histopathological characteristics of the study population. Since immune histochemical (IHC) analyses of the routine markers ER, PR, HER2, KI67 and p53 were not yet standardized in 1989–1993, we have re-evaluated these markers based on the current IHC methodology [10,11]. The molecular subtypes of breast cancer were defined based on these IHC analyses as follows: triple negative, ER-, PR- and HER2-; HER2-type, ER-, PR- and HER2+; luminal A, ER+ and/or PR+, HER2-; luminal B, ER+ and/or PR+, HER2+; luminal, ER+ and/or PR+ [9]. Upon the completion of genotyping, several additional patients and control subjects were excluded from further analyses due to technical genotyping failure (see Section 2.2). Accordingly, all analyses shown for rs2046210 are based on the successful genotyping of 409 breast cancer patients and 422 controls (Table S1), as well as 408 breast cancer patients and 415 controls for rs9382590 (Table S2).

### 2.2. DNA Isolation and SNV Genotyping

Genomic DNA for genotyping was extracted with the QIAamp DNA Blood Midi Kit (Qiagen, Venlo, The Netherlands) from peripheral lymphocytes and with the High Pure PCR Template Preparation Kit (Roche, Vienna, Austria) from fresh-frozen tumor tissue, as described [12,13]. The DNA was dissolved in TE buffer and stored at −80 °C. The SNVs were genotyped on a CFX96 real-time PCR instrument (BioRad, Vienna, Austria) by TaqMan PCR with Genotyping Master Mix and allele-specific, fluorescently labeled probes (Assay-IDs: rs2046210, C__12034236_10; rs9383590, C_30470113_10) purchased from Applied Biosystems (Brunn am Gebirge, Austria), following the manufacturers’ instructions. The PCR reaction volumes were 10 µL, and 20 ng of genomic DNA was used as a template in each reaction. As quality control measures, (i) two to six negative controls (2 μL ddH_2_O instead of DNA) were included in each qPCR run; (ii) 14 (rs2046210) and 111 (rs9383590) samples were genotyped in duplicate, including five samples from two separate DNA isolations each, with 100% concordance; (iii) the patient, control and duplicate statuses were blinded to the laboratory staff. Genotyping failed for one (rs2046210) or two (rs9383590) patients and for two (rs2046210) or nine (rs9383590) controls.

### 2.3. Statistical Analyses

Statistical analyses were performed with R version 3.3.2, an open-source language and environment for statistical computing, available from www.r-project.org (accessed on 1 July 2019) [14]. SNV genotypes are categorical variables with three categories (the three genotypes) and were handled as such. Since the CC genotype of rs9383590 is rare, CC subjects were combined with TC subjects into one category and were compared to TT subjects as a second category in all analyses. For some analyses of rs2046210, two genotypes were analogously combined into one category and were compared to the third genotype as a second category. Routine clinical and histopathological categories of breast cancer were applied according to the current practice (e.g., ER, PR, HER2 pos vs. neg), as indicated in respective figures and tables. The Hardy–Weinberg equilibrium was evaluated by chi-square tests with Yates’ continuity correction. Confidence intervals and *p*-values associated with odds ratios were calculated by the mid-P exact method. We consider our subgroup analyses as exploratory and therefore did not adjust for multiple testing, as recommended [15]. Comparisons of continuous variables (e.g., age at onset) between groups were analyzed with Kruskal–Wallis tests. Follow-up details of our study population, including the numbers of events as well as the mean and median follow-up times, have been described [9,12]. Survival was analyzed by univariable Cox proportional hazards models and by the Kaplan–Meier method. *p*-values for Kaplan–Meier curves and for cumulative breast cancer incidences were determined by log-rank tests, as described [16]. All *p*-values shown are two-sided. Associations with *p*-values < 0.05 were considered statistically significant.

## 3. Results

### 3.1. Distribution of ESR1 rs2046210 and rs9383590 SNV Genotypes

Two single nucleotide variants (SNV; SNP) located in a distal enhancer region 5′ to the human estrogen receptor 1 (*ESR1*) gene (rs2046210 and rs9383590) were genotyped in a hospital-based case-control study of 409 breast cancer patients and 422 female controls. The clinical characteristics of the study population, together with the frequency of the rs2046210 and rs9383590 genotypes in the study population and its subpopulations, are shown in Tables S1 and S2. The physical distance between rs2046210 and rs9383590 is 5399 bp, with rs9383590 being closer to the *ESR1* gene. The two SNVs were in almost complete linkage disequilibrium in our study population (D’ = 0.9988995; r^2^ = 0.41; *p* ≈ 0). Notably, the rs9383590 CC genotype (*n =* 5) co-occurred only with the rs2046210 AA genotype, and the rs2046210 GG genotype (*n =* 346) co-occurred only with the rs9383590 TT genotype. The frequencies of the genotypes GG, GA and AA of rs2046210 were 0.389, 0.477 and 0.134 in patients and 0.443, 0.443 and 0.114 in controls (Table S1). The frequency of the minor A-allele (MAF) was 0.373 in patients and 0.335 in controls, similar to the global MAF of 0.359 and the MAF of 0.350 reported for Europeans by the NCBI allele frequency aggregator [17]. For rs9383590, the frequencies of the genotypes TT, TC and CC were 0.824, 0.167 and 0.01 in patients and 0.846, 0.152 and 0.002 in controls (Table S2). The MAF was 0.093 in patients and 0.078 in controls, close to the global MAF of 0.057 and the MAF of 0.064 reported for Europeans [17]. The control population (*p* = 0.96 for rs2046210; *p* = 0.46 for rs9383590) and the patient population (*p* = 0.75 for rs2046210; *p* = 0.96 for rs9383590) were in Hardy–Weinberg equilibrium for both SNVs.

### 3.2. Association of rs2046210 with Breast Cancer Susceptibility

To assess the breast cancer risk associated with rs2046210, odds ratios (OR), 95% confidence intervals (CI) and *p*-values were determined (Table 1). This analysis revealed moderately elevated odds ratios associated with the minor A-allele, which is consistent with previous reports [1,2,3,4,18,19]. However, none of the genotypes or alleles of rs2046210 were associated with a significantly increased breast cancer risk (e.g., per-allele OR, 1.18; 95% CI, 0.96–1.44; *p* = 0.108). Adjusting for age had only a minor effect on the strength of the observed associations (data not shown). We next tested whether rs9383590 could be the causal SNV responsible for the association of rs2046210 with breast cancer risk. Accordingly, we determined the ORs, 95% CIs and *p*-values for the rs2046210 genotypes and alleles adjusted for the rs9383590 genotype and in subjects with the rs9383590 common homozygote genotype only (TT; i.e., excluding subjects with the rs9383590 TC or CC genotype from this analysis). However, the observed association trends did not disappear in this analysis (which would have been indicated by ORs close to unity), and some of the ORs even increased in magnitude, indicating that rs9383590 is not the causal SNV responsible for the association of rs2046210 with breast cancer risk (Table 1).

### 3.3. Association of rs9383590 with Breast Cancer Susceptibility

rs9383590 has been suggested to be a causal SNV for the observed associations of polymorphisms in and around the *ESR1* locus with an increased breast cancer risk [7]. However, an analysis of the association of rs9383590 itself with breast cancer risk has not been reported thus far. Accordingly, odds ratios (OR), 95% confidence intervals (CI) and *p*-values for meaningful comparisons of the rs9383590 genotypes and alleles in our study population were determined (Table 2). Since the CC genotype was very rare (four patients and one control), this genotype was not analyzed separately, but only upon grouping together with the TC genotype in this and all subsequent analyses.

Similar to rs2046210 (see 3.2.), this analysis revealed moderately but non-significantly elevated odds ratios associated with the minor C-allele (e.g., per-allele OR, 1.21; 95% CI, 0.86–1.72; *p* = 0.278). Unlike for rs2046210, adjusting for age considerably increased the strength of the observed associations for rs9383590 (e.g., per-allele OR, 1.32; 95% CI, 0.87–2.00; *p* = 0.191; Table 2; see also Table S5). We next determined the ORs, 95% CIs and *p*-values for rs9383590 genotypes and alleles adjusted for the rs2046210 genotype; an analysis in subjects with the rs2046210 common homozygote genotype only was not feasible, since they all exhibited the rs9383590 genotype TT. Adjusting for the rs2046210 genotype resulted in an almost complete loss of the associations for rs9383590 (indicated by ORs close to unity), indicating that the observed association trends for rs9383590 are indirect effects of its strong linkage disequilibrium with rs2046210 and its association with breast cancer susceptibility (Table 2).

### 3.4. Association of rs9383590 and rs2046210 with Breast Cancer Susceptibility in Subpopulations

We next explored the potential associations of rs9383590 and rs2046210 with breast cancer risk in clinically, histologically and molecularly relevant subpopulations and determined per-allele ORs (rs9383590, T vs. C; rs2046210, A vs. G; Table 3). In contrast to unselected patients (Table 1 and Table 2), the odds ratios were significantly elevated in specific subpopulations: in patients with an age under 55 years, rs9383590 C vs. T (OR, 1.66; 95% CI, 1.07–2.56; *p* = 0.025; Table 3) and CC + TC vs. TT (OR, 1.65; 95% CI, 1.04–2.61; *p* = 0.035; Table S4).

The odds ratios for rs2046210 were also considerably elevated in patients aged under 55 years at borderline significance: A vs. G (OR, 1.29; 95% CI, 0.99–1.69; *p* = 0.058; Table 3) and AA vs. GG (OR, 1.66; 95% CI, 0.94–2.94; *p* = 0.083; Table S3). Consistently, ORs were also elevated in pre-menopausal patients for both SNVs: rs9383590 C vs. T (OR, 1.55; 95% CI, 0.92–1.60; *p* = 0.108); rs2046210 A vs. G (OR, 1.33; 95% CI, 0.97–1.82; *p* = 0.081; Table 3).

### 3.5. Association of rs9383590 and rs2046210 with the Breast Cancer Onset Age

**Figure 1:**
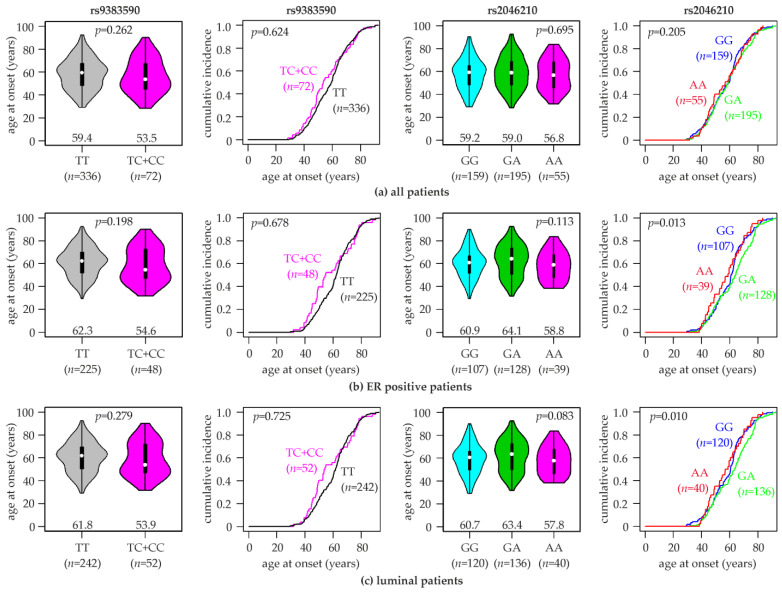
Association of rs9383590 and rs2046210 genotypes with the age at breast cancer onset. Violin plots (first and third column) and curves of the cumulative breast cancer incidence (second and fourth column) with the indicated age of onset are shown for (**a**) all patients in the study population, (**b**) ER-positive patients and (**c**) luminal patients. rs9383590 genotypes (TT, TC + CC; left half), rs2046210 genotypes (GG, GA, AA; right half) and numbers of patients (*n*) are indicated. Numbers in violin plots represent the median age of breast cancer onset for each genotype (indicated by white dots). *p*-values (*p*) to violin plots were calculated with Kruskal–Wallis tests, and those to curves of the cumulative breast cancer incidence were calculated with log-rank tests.

Since rs9383590 and rs2046210 were associated with a considerably increased breast cancer risk in women with an age under 55 years, we investigated their potential impact on the age at breast cancer onset. We found that patients with the rs9383590 TC + CC genotypes were considerably more likely to be diagnosed with breast cancer at an age under 55 years, as indicated by a steeper curve of the cumulative breast cancer incidence (Figure 1). However, the curves of TC + CC and of TT patients realigned at an age of breast cancer onset >60 years. The mean age of breast cancer onset for patients with the rs9383590 TC + CC genotypes was 56.5 ± 14.8 years (median, 53.5), and that of TT patients was 58.4 ± 13.4 years (median, 59.4; Figure 1). Thus, the TC + CC genotypes were non-significantly associated with a 5.9-years-younger median age at onset compared to the TT genotype (*p* = 0.26, Kruskal–Wallis test). These differences in the mean age of onset and in the curves of cumulative breast cancer incidence were more pronounced in ER-positive patients and in luminal patients (Figure 1). In ER-positive patients, the median age of breast cancer onset associated with the rs9383590 TC + CC genotypes was 54.6 years (mean, 58.7 ± 15.0 years), and that of TT patients was 62.3 years (mean, 61.5 ± 12.6 years; Figure 1), resulting in a 7.7-years-younger median age and a 2.8-years-younger mean age of ER-positive TC + CC patients (*p* = 0.198; Figure 1). In luminal A patients, we found the following median ages of breast cancer onset for rs9383590 genotypes: TC + CC, 53.9 years (mean, 58.5 ± 15.7 years), and TT, 61.8 years (mean, 60.6 ± 13.0 years; *p* = 0.279; Figure 1). The majority of luminal patients in our study populations exhibited the luminal A subtype (234/294). Similar but less pronounced differences in the age of onset were observed in luminal A compared to luminal patients (Figure S1). In contrast, the characteristic steeper curve of the cumulative breast cancer incidence under 55 years of TC + CC patients was not observed in ER-negative patients, and rs9383590 had almost no impact on the age of onset in this subpopulation (*p* = 0.931; Figure S1).

Analogous analyses for rs2046210 revealed a similar pattern of association. The rs2046210 genotype was significantly associated with the cumulative incidence of breast cancer by age in ER-positive (*p* = 0.013, log-rank test), luminal (*p* = 0.010) and luminal A patients (*p* = 0.022), but not in unselected (*p* = 0.205) or ER-negative patients (*p* = 0.164; Figure 1 and Figure S1). Similar to rs9383590, patients with the minor homozygous genotype of rs2046210 (AA) exhibited a younger median and mean age at breast cancer onset compared to those with the other two genotypes. The following median ages of onset by the rs2046210 genotype were observed: in ER-positives, AA, 58.8 years (mean, 58.1 ± 12.4 years) vs. GG + GA, 62.3 years (mean, 61.5 ± 13.1 years; *p* = 0.12; Kruskal–Wallis test); in luminal patients, AA, 57.8 years (mean, 57.8 ± 12.4 years) vs. GG + GA, 61.8 years (mean, 60.6 ±13.4 years; *p* = 0.19); in luminal A patients, AA, 59.2 years (mean, 59.2 ± 12.7 years) vs. GG + GA, 62.7 years (mean, 61.8 ± 13.4 years; *p* = 0.28). We next analyzed the association of rs2046210 with the age at breast cancer onset in patients with the common homozygous genotype of rs9383590 (TT; Figure S1). In this subset, all three rs2046210 genotypes exhibited very similar median (GG, 59.2; GA, 59.2; AA, 59.6 years) and mean ages at onset (GG, 57.4 ± 13.4; GA, 59.3 ± 13.4; AA, 58.9 ± 13.8 years; *p* = 0.558), indicating that the earlier breast cancer onset of AA patients unselected for the rs9383590 genotype was an indirect effect of the linkage disequilibrium with rs9383590. Notably, 22 of the 55 rs2046210 AA patients exhibited the rs9383590 genotypes TC or CC (40%). A reciprocal analysis of rs9383590 genotypes in rs2046210 common homozygotes was not feasible, since they all exhibited the same rs9383590 genotype in our study population (TT).

### 3.6. Association of rs9383590 and rs2046210 with Breast Cancer Prognosis

The association of the rs9383590 and rs2046210 genotypes with the overall (OS), disease-free (DFS) and metastasis-free survival (MFS) was assessed in a Cox proportional hazards analysis and in a Kaplan–Meier analysis of 134 patients and in subsets thereof. Collectively, our survival analysis revealed that the minor risk genotypes/alleles of both SNVs tended to be associated with a favorable prognosis in unselected, ER-positive and luminal A patients, but not in ER-negative patients (Table 4 and Table S6; Figures S2 and S3). In ER-negative patients, the rs2046210 AA genotype tended to be non-significantly associated with a poor prognosis (Figure S3), whereas no consistent trend was observed for rs9383590 (Figure S2). When all three genotypes were examined as separate categories, rs2046210 was significantly associated with the MFS of luminal A patients (*p* = 0.037, Cox proportional hazards analysis; *p* = 0.031, log-rank test; Table S6 and Figure S3). Herein, the GG genotype was associated with the poorest prognosis, and the AA genotype was associated with the most favorable prognosis (Figure S3). A borderline significance was observed when rs2046210 GA + AA genotypes were grouped together and compared to the GG genotype (HR, 0.46; 95% CI, 0.21-1.01; *p* = 0.053; Table 4). In general, the strongest associations with the prognosis for both SNVs were observed in luminal A patients (Table 4 and Table S6; Figures S2 and S3). However, even though the rs2046210 AA and rs9383590 TC + CC genotypes tended to be associated with a favorable prognosis in these other analyses as well, no further associations were significant at the *p* < 0.05 level (Table 4 and Table S6, Figures S2 and S3). 

## 4. Discussion

The *ESR1* gene encoding ERα is perhaps the most clinically relevant gene in breast cancer biology, with key roles as a target of systemic therapy, a predictive and prognostic marker and a determinant of the molecular subtype [20]. Arguably, breast cancer treatment with the selective estrogen receptor modulator tamoxifen was the first targeted therapy of cancer [21]. Accordingly, a thorough understanding of the regulation of *ESR1* expression is of utmost clinical and scientific importance. Here, we analyzed the association of two noncoding SNVs in a putative enhancer region upstream of the *ESR1* gene with the risk, age at onset, clinically and molecularly relevant characteristics and prognosis of breast cancer. Evidence has accumulated indicating that genetic variants in and around the *ESR1* locus are associated with various diseases and phenotypes in which estrogen and the estrogen receptor play a role, such as age at menarche, mammographic density, breast cancer, endometrial cancer, endometriosis and bone mineral density [22,23,24,25,26,27,28]. Several of these variants appear to be located in putative enhancer regions and to play a role in the regulation of *ESR1* expression [4,7]. 

We found that the minor alleles of both rs2046210 and rs9383590 tend to be associated with an increased breast cancer risk to a similar extent and in largely overlapping breast cancer subtypes. The minor allele of rs9383590 was significantly associated with an increased breast cancer risk in patients under 55 years, and the minor allele of rs2046210 showed the same association but at borderline significance. Moreover, rs2046210 was significantly associated with the risk of PR-negative breast cancer. In contrast, no consistent trend of association was observed in ER-positive vs. -negative disease for either of the SNVs. The minor homozygous genotype of both SNVs (AA of rs2046210; CT + CC of rs9383590) tended to be associated with a several-years-earlier age at breast cancer onset, which was more pronounced in ER-positive and luminal subtypes. An association with both an increased risk and an earlier age at onset is a common observation for high-penetrance genetic risk factors such as mutations in *BRCA1* or *BRCA2*. This is, however, not universally observed for common low-penetrance genetic polymorphisms such as rs2046210 and rs9383590.

Cox proportional hazards analysis and Kaplan–Meier analysis revealed that the risk alleles of both SNVs tended to be associated with a favorable prognosis in unselected, ER-positive and luminal A patients, but not in ER-negative patients. It may appear unexpected that risk genotypes or alleles tend to be associated with a favorable rather than a poor prognosis. However, it was repeatedly demonstrated that genetic variants associated with breast cancer susceptibility are rarely associated in the same way with breast cancer prognosis [29,30,31,32,33]. Consistent with our results, the GA genotype of rs2046210 was previously shown to be associated with a favorable OS, DFS and MFS of patients with early breast cancer [33]. The association of the risk alleles of rs2046210 and rs9383590 with a favorable prognosis could be mechanistically explained by the potential impact of these SNVs on *ESR1* expression. The rs2046210 AA genotype has been reported to be associated with a significantly lower ERα expression than the GG genotype [4]. ERα is a potent driver of the proliferation and progression of ER-positive breast cancer, and, thus, this lower expression of *ESR1*/ERα may lead to a less aggressive tumor progression and, hence, a favorable prognosis. Consistent with this hypothesis, we observed the association of the rs2046210A and rs9383590C alleles with a favorable prognosis only in ER-positive patients and in the hormone receptor-positive luminal A molecular subtype, and not in ER-negative disease, where ERα does not exhibit this tumor-promoting effect.

Evidence for a functional role of rs9383590 has been reported: rs9383590 is located in a binding site of the transcription factor GATA3, its minor allele (C) binds GATA3 with a lower affinity than the common T-allele and the deletion of a 2.2 kb region containing rs9383590 reduced the expression of *ESR1* [7]. Likewise, the minor A-allele of rs2046210 was found to be associated with a reduced expression of *ESR1* [4]. Thus, both SNVs were independently identified as causal candidates in the same putative upstream enhancer region in two separate studies [4,7]. Accordingly, we aimed at a systematic, side-by-side comparison of both SNVs in the same study population to address the question of whether rs9383590 is the functional SNV causing the observed associations of rs2046210 (or vice versa). Our approach was to determine the impact of adjusting for one SNV, and of analyses in the common homozygotes of that SNV, on the strength of associations with the other one, thus eliminating any potential confounding effect of one SNV on the associations of the other. If rs9383590 was the causal variant responsible for the associations observed for rs2046210, this approach would nullify these associations for rs2046210 (and vice versa).

We found that the elevated odds ratios with respect to the breast cancer risk of rs2046210 genotypes and alleles were not substantially lessened upon correction for rs9383590 or upon analysis in rs9383590 common homozygotes (TT; Table 1). In contrast, adjusting for the rs2046210 genotype resulted in an almost complete loss of the observed association trend in unselected patients for rs9383590 (as indicated by substantially lessened ORs close to unity; Table 2), suggesting that these trends are indirect effects of its strong linkage disequilibrium with rs2046210. However, the significant association of rs9383590 with an increased breast cancer risk in patients under 55 years remained elevated upon adjusting for the rs2046210 genotype (data not shown). Moreover, in patients with the common homozygous genotype of rs9383590 (TT), all three rs2046210 genotypes exhibited very similar mean and median ages at onset, indicating that the earlier onset of AA patients unselected for the rs9383590 genotype was an indirect effect of rs9383590 (Figure S1c). A reciprocal analysis of rs9383590 genotypes in rs2046210 common homozygotes was precluded by the finding that none of them exhibited the rs9383590 genotypes TC or CC in our study population. Collectively, our findings are not consistent with a model in which rs9383590 is the only causal SNV in region 1 responsible for the association of genetic variants in this region with breast cancer risk. Rather, rs2046210 is either an additional causal variant itself or associated with another causal variant in this region in addition to rs9383590. Importantly, rs2046210 emerged as the top causal candidate in region 1, but nine additional SNVs could not be excluded as causal candidates in this region in a large genetic epidemiological study [4].

A possible limitation of our study is its moderate size, particularly since follow-up information was only available for 134 out of the 410 patients. However, the effective population size in survival analyses is determined by the number of events, ameliorating these size limitations. The regulation of ESR1 appears to be substantially modified by several genetic variants associated with breast cancer susceptibility, and we have analyzed two of the top causal candidates in an upstream enhancer region, each identified in independent study populations and experimental systems [4,7], in a systematic side-by-side comparison. Our results revealed that the minor alleles of rs2046210 and rs9383590 were both associated with an increased breast cancer risk, an earlier age at onset and a favorable prognosis to a similar extent and in largely overlapping breast cancer subtypes.

## Figures and Tables

**Table 1:** Association of *ESR1* rs2046210 genotypes and alleles with breast cancer risk.

rs2046210 Genotypes or Alleles	All Subjects		All Subjects		rs9383590 TT Subjects	
	Unadjusted		Adjusted for rs9383590		Unadjusted	
	OR (95% CI)	*p*	OR (95% CI)	*p*	OR (95% CI)	*p*
AA vs. GG	1.35 (0.87–2.09)	0.184	1.21 (0.89–1.65)	0.335	1.53 (0.87–2.71)	0.139
AA vs. GA	1.10 (0.71–1.69)	0.672	1.23 (0.91–1.66)	0.172	1.32 (0.74–2.33)	0.351
AA vs. GA + GG	1.21 (0.80–1.83)	0.365	1.17 (0.93–1.46)	0.174	1.43 (0.82–2.48)	0.202
GA vs. GG	1.23 (0.92–1.64)	0.170	1.32 (0.81–2.14)	0.103	1.17 (0.85–1.60)	0.335
AA + GA vs. GG	1.25 (0.95–1.65)	0.112	1.08 (0.68–1.69)	0.172	1.22 (0.90–1.65)	0.194
A vs. G	1.18 (0.96–1.44)	0.108	1.16 (0.75–1.81)	0.502	1.21 (0.95–1.53)	0.117

Analyses of breast cancer cases vs. controls of the indicated rs2046210 genotypes or alleles. Analyses were conducted in all subjects or in subjects with the TT genotype of the *ESR1* rs9383590 SNV only, unadjusted or adjusted for the rs9383590 genotype, as indicated. OR, odds ratios; 95% CI, 95% confidence intervals; *p*, *p*-values.

**Table 2:** Association of *ESR1* rs9383590 genotypes and alleles with breast cancer risk.

Genotypes or Alleles	Unadjusted		Adjusted for Age		Adjusted for rs2046210	
	OR (95% CI)	*p*	OR (95% CI)	*p*	OR (95% CI)	*p*
TC vs. TT	1.13 (0.78–1.64)	0.529	1.25 (0.80–1.96)	0.324	1.01 (0.67–1.52)	0.956
TC + CC vs. TT	1.18 (0.81–1.70)	0.390	1.30 (0.84–2.01)	0.246	1.04 (0.70–1.56)	0.832
C vs. T	1.21 (0.86–1.72)	0.278	1.32 (0.87–2.00)	0.191	1.09 (0.74–1.61)	0.648

Analyses of breast cancer cases vs. controls of the indicated rs9383590 genotypes or alleles. Analyses were conducted in all subjects, unadjusted, adjusted for age or adjusted for rs2046210 genotype, as indicated. OR, odds ratios; 95% CI, 95% confidence intervals; *p*, *p*-values.

**Table 3:** Association of rs938359s0 and rs2046210 with breast cancer risk in patient subpopulations.

Category	Subcategory	Patients		rs9383590 (C vs. T)		rs2046210 (A vs. G)	
		No.	%	OR (95% CI)	*p*	OR (95% CI)	*p*
Age *	<55	173	42.3%	**1.66 (1.07**–**2.56)**	**0.025**	1.29 (0.99–1.69)	0.058
	≥55	236	57.7%	0.81 (0.39–1.68)	0.600	0.91 (0.59–1.40)	0.665
Age **	<55	173	42.3%	**1.61 (1.06**–**2.47)**	**0.029**	1.25 (0.97–1.62)	0.090
	≥55	236	57.7%	0.95 (0.61–1.46)	0.800	1.13 (0.89–1.43)	0.322
Menopausal status	pre	101	28.6%	1.55 (0.92–1.60)	0.108	1.33 (0.97–1.82)	0.081
	post	252	71.4%	1.13 (0.76–1.69)	0.547	1.15 (0.91–1.45)	0.235
Tumor type	ductal	250	75.5%	1.34 (0.91–1.98)	0.135	1.14 (0.91–1.44)	0.257
	lobular	81	24.5%	1.12 (0.60–1.09)	0.680	1.14 (0.80–1.61)	0.479
Tumor size	pT1	176	52.4%	1.07 (0.67–1.70)	0.789	1.17 (0.91–1.51)	0.231
	pT2-4	160	47.6%	1.46 (0.94–1.28)	0.095	1.14 (0.87–1.50)	0.329
Stage	0 or I	144	43.4%	0.83 (0.49–1.43)	0.500	1.07 (0.81–1.42)	0.634
	II-IV	188	56.6%	1.42 (0.93–1.17)	0.109	1.18 (0.91–1.52)	0.210
Grade	pG1-2	240	62.2%	1.05 (0.68–1.61)	0.834	1.21 (0.96–1.53)	0.110
	pG3	146	37.8%	1.51 (0.96–1.35)	0.077	1.21 (0.91–1.60)	0.194
Lymph node status	pN0	197	59.7%	1.01 (0.64–1.58)	0.963	1.19 (0.93–1.53)	0.165
	pN+	133	40.3%	1.52 (0.95–1.41)	0.086	1.05 (0.79–1.40)	0.754
ER status	pos	274	69.7%	1.17 (0.79–1.74)	0.441	1.19 (0.95–1.49)	0.123
	neg	119	30.3%	1.26 (0.76–1.09)	0.369	1.12 (0.83–1.52)	0.452
PR status	pos	188	48.7%	1.07 (0.67–1.70)	0.804	1.07 (0.83–1.39)	0.591
	neg	198	51.3%	1.29 (0.85–1.96)	0.239	**1.29 (1.01–1.66)**	**0.044**
HER2 status	pos	74	19.9%	1.14 (0.60–1.18)	0.663	1.24 (0.87–1.78)	0.244
	neg	298	80.1%	1.18 (0.81–1.73)	0.394	1.18 (0.95–1.47)	0.144
KI67 status (%pos cells)	>10%	125	40.5%	1.20 (0.72–1.98)	0.490	1.00 (0.74–1.35)	0.984
	≤10%	184	59.5%	1.09 (0.69–1.73)	0.703	1.25 (0.97–1.61)	0.091
TP53 status	pos	98	25.6%	1.67 (1.00–1.76)	0.055	1.21 (0.87–1.67)	0.262
	neg	285	74.4%	1.06 (0.71–1.59)	0.759	1.22 (0.98–1.52)	0.081
Molecular subtype	luminal	296	82.7%	1.17 (0.79–1.73)	0.427	1.14 (0.91–1.42)	0.249
	HER2 type	33	9.2%	1.43 (0.61–1.37)	0.378	1.29 (0.77–2.15)	0.339
	triple neg	62	17.3%	1.26 (0.66–1.41)	0.491	1.30 (0.88–1.93)	0.190

Per-allele odds ratios (log-additive model) of the minor vs. the common allele of rs9383590 and rs2046210 in the indicated clinically and molecularly relevant subcategories of breast cancer are shown. Significant associations are highlighted in bold. OR, odds ratios; 95% CI, 95% confidence intervals; *p*, *p*-values; No., number of patients in the indicated subcategories (numbers are for rs 2046210; one patient fewer was genotyped for rs9383590; see Table S4); %, fraction of patients in the indicated subcategories (in %); pre, pre-menopausal; post, post-menopausal; ER, estrogen receptor; PR, progesterone receptor; pos, positive; neg, negative. * cases aged <55 or ≥55 years vs. controls aged <55 or ≥55 years; ** cases aged <55 or ≥55 years vs. controls of any age (for the sake of comparability with the other subcategory analyses).

**Table 4:** Univariable analyses of the overall, disease-free and metastasis-free survival using a Cox proportional hazards model.

Survival Type	Patients	rs9383590		rs2046210	
		HR (95% CI)	*p*	HR (95% CI)	*p*
Overall survival	all	0.95 (0.55–1.66)	0.867	0.99 (0.62–1.57)	0.956
	ER pos	1.01 (0.47–2.19)	0.978	0.96 (0.53–1.77)	0.906
	ER neg	0.81 (0.36–1.81)	0.609	0.90 (0.43–1.87)	0.774
	luminal A	0.67 (0.26–1.75)	0.417	0.80 (0.41–1.56)	0.508
Disease-free survival	all	0.85 (0.47–1.52)	0.574	0.92 (0.58–1.47)	0.731
	ER pos	0.73 (0.31–1.75)	0.487	0.93 (0.50–1.73)	0.820
	ER neg	0.81 (0.36–1.81)	0.610	0.81 (0.40–1.66)	0.563
	luminal A	0.41 (0.12–1.34)	0.140	0.64 (0.31–1.31)	0.224
Metastasis-free survival	all	0.86 (0.46–1.63)	0.651	0.87 (0.53–1.45)	0.603
	ER pos	0.70 (0.27–1.82)	0.469	0.78 (0.40–1.50)	0.452
	ER neg	0.95 (0.40–2.25)	0.899	0.98 (0.43–2.19)	0.953
	luminal A	0.32 (0.08–1.35)	0.121	0.46 (0.21–1.01)	0.053

Analyses of the overall survival, disease-free survival and metastasis-free survival were conducted in all patients, ER-pos patients, ER-neg patients and luminal A patients, as indicated. Genotypes were coded as follows: rs9383590, TT = 0 (common homozygote genotype), TC = 1, CC = 1; rs2046210, GG = 0 (common homozygote genotype), GA = 1, AA = 1. HR, hazard ratios; 95% CI, 95% confidence intervals; *p*, *p*-values; ER, estrogen receptor; pos, positive; neg, negative. Hazard ratios < 1 indicate a favorable prognosis associated with the risk genotypes (coded as 1).

## Data Availability

The data presented in this study are available in the article and the supplementary materials.

## Supplementary Materials

The following supporting information can be downloaded at: https://www.mdpi.com/article/10.3390/cells12040515/s1, Figure S1: Association of rs9383590 and rs2046210 genotypes with the age at breast cancer onset in additional subpopulations; Figure S2: Association of rs9383590 genotypes with the survival of human breast cancer patients; Figure S3: Association of rs2046210 genotypes with the survival of human breast cancer patients; Table S1: Clinical characteristics of the study population, and genotype frequencies of rs2046210; Table S2: Clinical characteristics of the study population, and genotype frequencies of rs9383590; Table S3: Additional analyses of the association of rs2046210 with breast cancer risk in patient subpopulations; Table S4: Additional analyses of the association of rs9383590 with breast cancer risk in patient subpopulations; Table S5: Age-adjusted analyses of the association of rs9383590 with breast cancer risk in patient subpopulations; Table S6: Univariable analyses of the overall, disease-free and metastasis-free survival using a Cox proportional hazards model.
