# Supplementary material for: Association of the Estrogen Receptor 1 Polymorphisms rs2046210 and rs9383590 with the Risk, Age at Onset and Prognosis of Breast Cancer
Source: Cells. 2023 Feb 4;12(4):515. doi: 10.3390/cells12040515 (PMC9953811; doi:10.3390/cells12040515)
Supplement: Supplementary file 1 [file cells-12-00515-s001.zip › Figure S1.pdf]

cells

MDPI

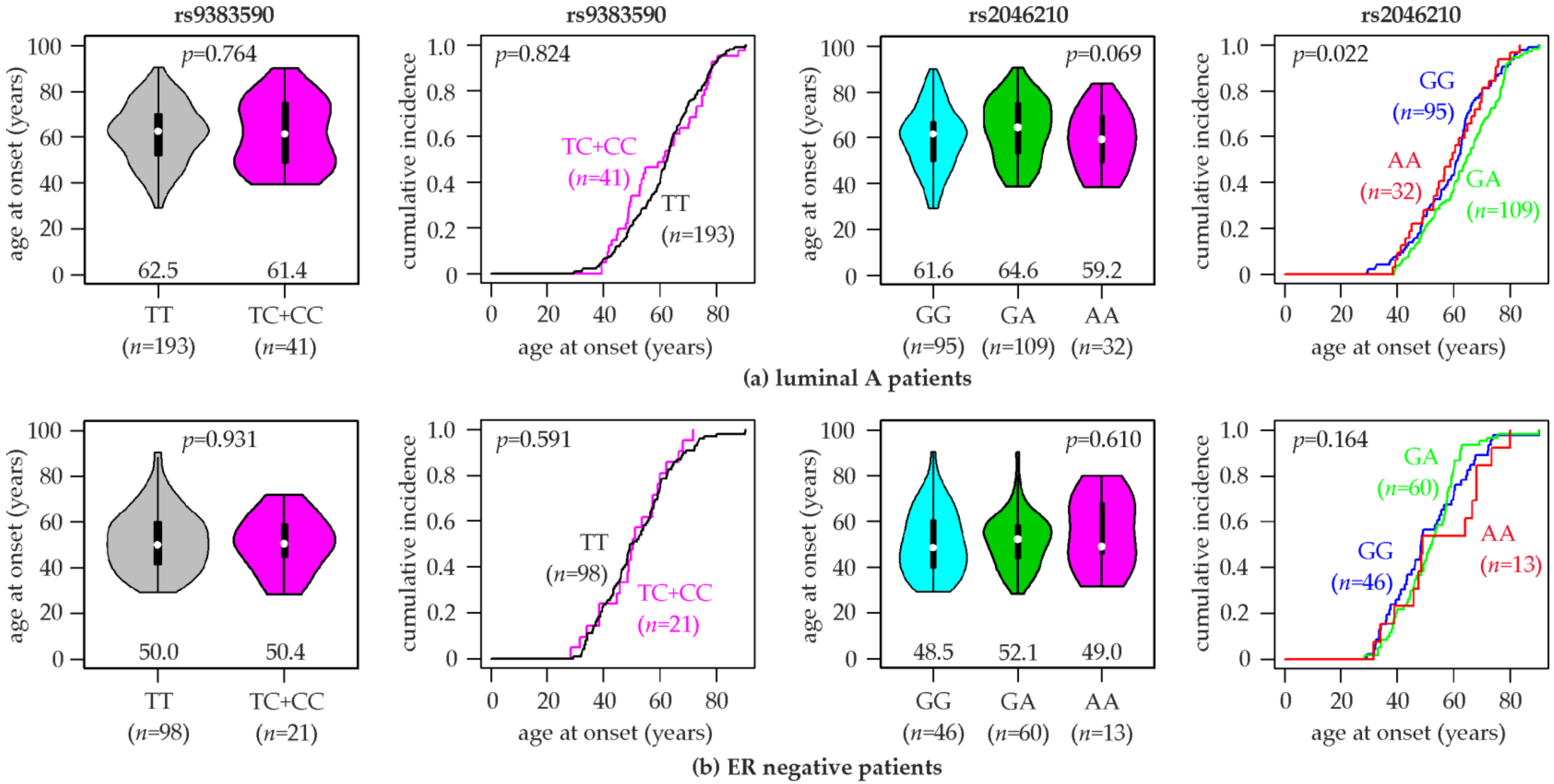

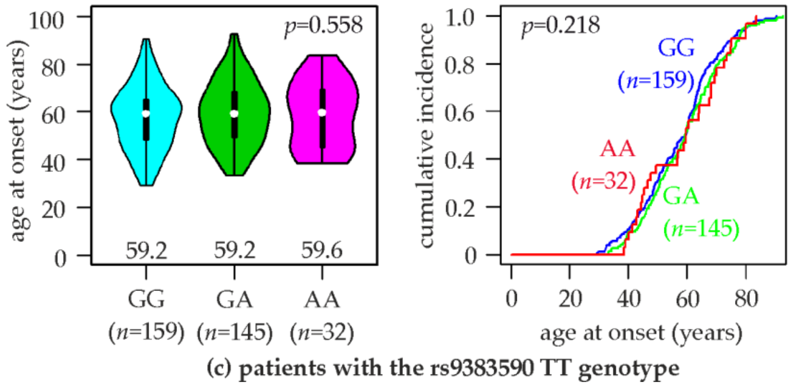

Figure S1. Association of rs9383590 and rs2046210 genotypes with the age at breast cancer onset in additional subpopulations. Violin plots (first and third column) and curves of the cumulative breast cancer incidence (second and fourth column) with the indicated age of onset are shown for (a) luminal A patients, (b) ER negative patients, and (c) patients with the rs9383590 TT genotype. rs9383590 genotypes, rs2046210 genotypes and numbers of patients (*n*) are indicated. Numbers in violin plots represent the median age of breast cancer onset for each genotype. *p*-values (*p*) to violin plots were calculated with Kruskal-Wallis tests, and to curves of the cumulative breast cancer incidence with log-rank tests.
