# Supplementary material for: Association of the Estrogen Receptor 1 Polymorphisms rs2046210 and rs9383590 with the Risk, Age at Onset and Prognosis of Breast Cancer
Source: Cells. 2023 Feb 4;12(4):515. doi: 10.3390/cells12040515 (PMC9953811; doi:10.3390/cells12040515)
Supplement: Supplementary file 1 [file cells-12-00515-s001.zip › Figure S2.pdf]

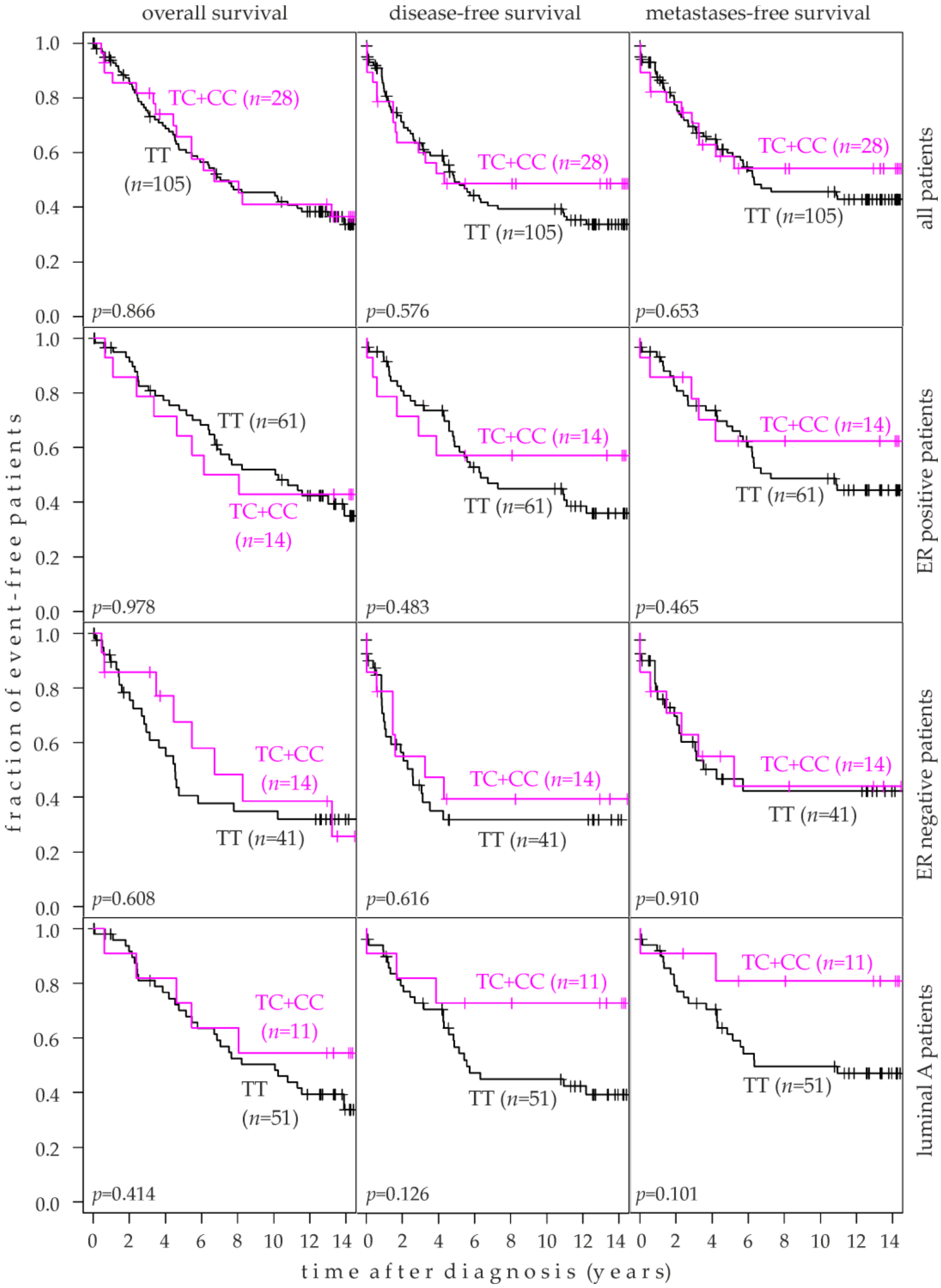

Figure S2. Association of rs9383590 genotypes with the survival of human breast cancer patients. Patients with TC and CC genotypes were combined into one group, and compared to TT patients. Kaplan-Meier analyses of the overall survival, disease-free survival and metastasis-free survival in unselected patients (top row; *n*=133), estrogen receptor (ER) positive patients (second row; *n*=75), ER negative patients (third row; *n*=55), and luminal A patients (bottom row; *n*=62) are shown. Numbers (*n*) of patients in each group and *p*-values (*p*) determined by log-rank tests are indicated.
