# Supplementary material for: Association of the Estrogen Receptor 1 Polymorphisms rs2046210 and rs9383590 with the Risk, Age at Onset and Prognosis of Breast Cancer
Source: Cells. 2023 Feb 4;12(4):515. doi: 10.3390/cells12040515 (PMC9953811; doi:10.3390/cells12040515)
Supplement: Supplementary file 1 [file cells-12-00515-s001.zip › Figure S3.pdf]

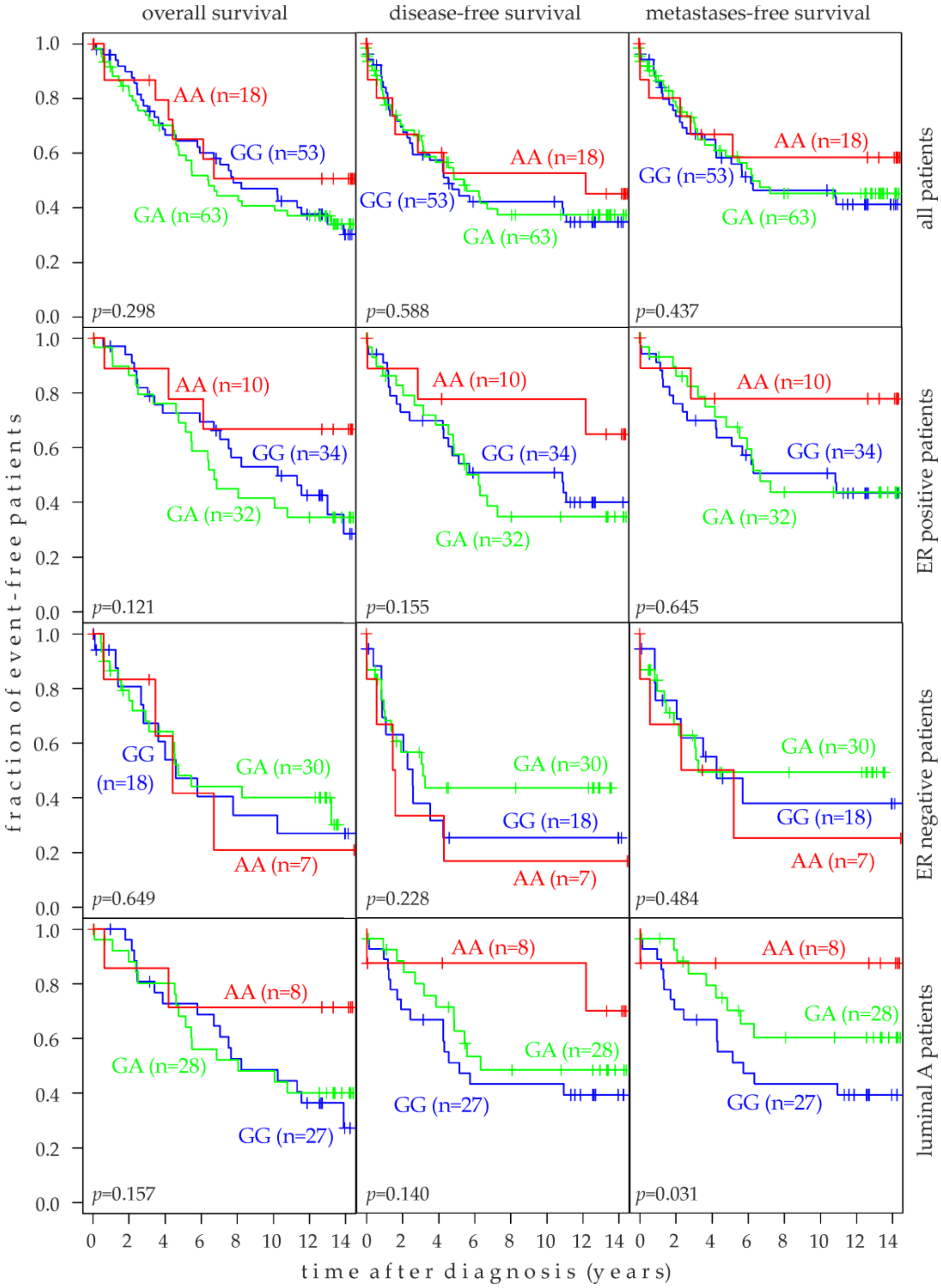

Figure S3. Association of rs2046210 genotypes with the survival of human breast cancer patients. Kaplan-Meier analyses of the overall survival, disease-free survival and metastasis-free survival in unselected patients (top row; *n*=134), estrogen receptor (ER) positive patients (second row; *n*=76), ER negative patients (third row; *n*=55), and luminal A patients (bottom row; *n*=63) are shown. Numbers (*n*) of patients in each group and *p*-values (*p*) determined by log-rank tests are indicated.
