# Supplementary material for: Association of the Estrogen Receptor 1 Polymorphisms rs2046210 and rs9383590 with the Risk, Age at Onset and Prognosis of Breast Cancer
Source: Cells. 2023 Feb 4;12(4):515. doi: 10.3390/cells12040515 (PMC9953811; doi:10.3390/cells12040515)
Supplement: Supplementary file 1 [file cells-12-00515-s001.zip › Table S1.pdf]

Table S1. Clinical characteristics of the study population, and genotype frequencies of rs2046210.

		Total	GG	GA	AA
All subjects		831	346 (41.6%)	382 (46.0%)	103 (12.4%)
Patients		409	159 (38.9%)	195 (47.7%)	55 (13.4%)
Controls		422	187 (44.3%)	187 (44.3%)	48 (11.4%)
**Patient subgroups**					
Age (years)	<55	173	65 (37.6%)	82 (47.4%)	26 (15.0%)
	≥55	236	94 (39.8%)	113 (47.9%)	29 (12.3%)
Menopausal status	pre	101	36 (35.6%)	49 (48.5%)	16 (15.8%)
	post	252	99 (39.3%)	121 (48.0%)	32 (12.7%)
	na	56	24 (42.9%)	25 (44.6%)	7 (12.5%)
Tumor size	pT1	176	73 (41.5%)	75 (42.6%)	28 (15.9%)
	pT2-4	160	62 (38.8%)	79 (49.4%)	19 (11.9%)
	other, na	73	24 (32.9%)	41 (56.2%)	8 (11.0%)
Tumor type	ductal	250	102 (40.8%)	113 (45.2%)	35 (14.0%)
	lobular	81	32 (39.5%)	39 (48.1%)	10 (12.3%)
	other, na	78	25 (32.1%)	43 (55.1%)	10 (12.8%)
Stage	0-1	144	60 (41.7%)	67 (46.5%)	17 (11.8%)
	2-4	188	73 (38.8%)	90 (47.9%)	25 (13.3%)
	other, na	77	26 (33.8%)	38 (49.4%)	13 (16.9%)
Grade	pG1-2	240	93 (38.8%)	112 (46.7%)	35 (14.6%)
	pG3	146	52 (35.6%)	78 (53.4%)	16 (11.0%)
	na	23	14 (60.9%)	5 (21.7%)	4 (17.4%)
Lymph node status	pN0	197	75 (38.1%)	96 (48.7%)	26 (13.2%)
	pN+	133	59 (44.4%)	56 (42.1%)	18 (13.5%)
	na	68	25 (36.8%)	43 (63.2%)	0 (0.0%)
ER status	pos	274	107 (39.1%)	128 (46.7%)	39 (14.2%)
	neg	119	46 (38.7%)	60 (50.4%)	13 (10.9%)
	na	16	6 (37.5%)	7 (43.8%)	3 (18.8%)
PR status	pos	188	78 (41.5%)	88 (46.8%)	22 (11.7%)
	neg	198	71 (35.9%)	98 (49.5%)	29 (14.6%)
	na	23	10 (43.5%)	9 (39.1%)	4 (17.4%)
HER2 status	pos	74	28 (37.8%)	35 (47.3%)	11 (14.9%)
	neg	298	115 (38.6%)	144 (48.3%)	39 (13.1%)
	na	37	16 (43.2%)	16 (43.2%)	5 (13.5%)
TP53 status	pos	98	36 (36.7%)	50 (51.0%)	12 (12.2%)
	neg	285	109 (38.2%)	135 (47.4%)	41 (14.4%)
	na	26	13 (50.0%)	10 (38.5%)	3 (11.5%)
KI67 status (% pos cells)	≤10%	184	69 (37.5%)	88 (47.8%)	27 (14.7%)
	>10%	125	54 (43.2%)	58 (46.4%)	13 (10.4%)
	na	99	36 (36.4%)	48 (48.5%)	15 (15.2%)
Molecular subtype	luminal A	236	95 (40.3%)	109 (46.2%)	32 (13.6%)
	luminal B	41	16 (39.0%)	19 (46.3%)	6 (14.6%)
	HER2 type	33	12 (36.4%)	16 (48.5%)	5 (15.2%)
	triple neg	62	20 (32.3%)	35 (56.5%)	7 (11.3%)
	na	37	16 (43.2%)	16 (43.2%)	5 (13.5%)

Numbers of patients and controls, as well as number of patients in the indicated subgroups are shown. Parenthesized numbers show the fraction of patients (%) with the indicated genotypes. ER, estrogen receptor; PR, progesterone receptor; na, status not available.
