# Supplementary material for: Association of the Estrogen Receptor 1 Polymorphisms rs2046210 and rs9383590 with the Risk, Age at Onset and Prognosis of Breast Cancer
Source: Cells. 2023 Feb 4;12(4):515. doi: 10.3390/cells12040515 (PMC9953811; doi:10.3390/cells12040515)
Supplement: Supplementary file 1 [file cells-12-00515-s001.zip › Table S2.pdf]

cells MDPI

Table S2. Clinical characteristics of the study population, and genotype frequencies of rs9383590.

		Total	TT		TC		CC	
All subjects		823	687	(83.5%)	131	(15.9%)	5	(0.6%)
Patients		408	336	(82.4%)	68	(16.7%)	4	(1.0%)
Controls		415	351	(84.6%)	63	(15.2%)	1	(0.2%)
**Patient subgroups**								
Age (years)	<55	173	134	(77.5%)	37	(21.4%)	2	(1.2%)
	≥55	235	202	(86.0%)	31	(13.2%)	2	(0.9%)
Menopausal status	pre	101	79	(78.2%)	21	(20.8%)	1	(1.0%)
	post	251	210	(83.7%)	38	(15.1%)	3	(1.2%)
	na	56	47	(83.9%)	9	(16.1%)	0	(0.0%)
Tumor size	pT1	175	147	(84.0%)	27	(15.4%)	1	(0.6%)
	pT2-4	160	127	(79.4%)	31	(19.4%)	2	(1.3%)
	other, na	73	62	(84.9%)	10	(13.7%)	1	(1.4%)
Tumor type	ductal	249	202	(81.1%)	43	(17.3%)	4	(1.6%)
	lobular	81	67	(82.7%)	14	(17.3%)	0	(0.0%)
	other, na	78	67	(85.9%)	11	(14.1%)	0	(0.0%)
Stage	0-1	143	125	(87.4%)	17	(11.9%)	1	(0.7%)
	2-4	188	150	(79.8%)	36	(19.1%)	2	(1.1%)
	other, na	77	61	(79.2%)	15	(19.5%)	1	(1.3%)
Grade	pG1-2	239	200	(83.7%)	39	(16.3%)	0	(0.0%)
	pG3	146	116	(79.5%)	27	(18.5%)	3	(2.1%)
	na	23	20	(87.0%)	2	(8.7%)	1	(4.3%)
Lymph node status	pN0	196	167	(85.2%)	27	(13.8%)	2	(1.0%)
	pN+	133	105	(78.9%)	26	(19.5%)	2	(1.5%)
	na	79	64	(81.0%)	15	(19.0%)	0	(0.0%)
ER status	pos	273	225	(82.4%)	47	(17.2%)	1	(0.4%)
	neg	119	98	(82.4%)	19	(16.0%)	2	(1.7%)
	na	16	13	(81.3%)	2	(12.5%)	1	(6.3%)
PR status	pos	187	156	(83.4%)	31	(16.6%)	0	(0.0%)
	neg	198	162	(81.8%)	33	(16.7%)	3	(1.5%)
	na	23	18	(78.3%)	4	(17.4%)	1	(4.3%)
HER2 status	pos	74	61	(82.4%)	13	(17.6%)	0	(0.0%)
	neg	297	246	(82.8%)	48	(16.2%)	3	(1.0%)
	na	37	29	(78.4%)	7	(18.9%)	1	(2.7%)
TP53 status	pos	98	76	(77.6%)	20	(20.4%)	2	(2.0%)
	neg	284	238	(83.8%)	45	(15.8%)	1	(0.4%)
	na	26	13	(50.0%)	10	(38.5%)	3	(11.5%)
KI67 status (% pos cells)	≤10%	183	153	(83.6%)	29	(15.8%)	1	(0.5%)
	>10%	125	104	(83.2%)	19	(15.2%)	2	(1.6%)
	na	99	78	(78.8%)	20	(20.2%)	1	(1.0%)
Molecular subtype	luminal A	235	194	(82.6%)	40	(17.0%)	1	(0.4%)
	luminal B	41	35	(85.4%)	6	(14.6%)	0	(0.0%)
	HER2 type	33	26	(78.8%)	7	(21.2%)	0	(0.0%)
	triple neg	62	52	(83.9%)	8	(12.9%)	2	(3.2%)
	na	37	29	(78.4%)	7	(18.9%)	1	(2.7%)

Numbers of patients and controls, as well as number of patients in the indicated subgroups are shown. Parenthesized numbers show the fraction of patients (%) with the indicated genotypes. ER, estrogen receptor; PR, progesterone receptor; na, status not available.
