# Supplementary material for: Association of the Estrogen Receptor 1 Polymorphisms rs2046210 and rs9383590 with the Risk, Age at Onset and Prognosis of Breast Cancer
Source: Cells. 2023 Feb 4;12(4):515. doi: 10.3390/cells12040515 (PMC9953811; doi:10.3390/cells12040515)
Supplement: Supplementary file 1 [file cells-12-00515-s001.zip › Table S3.pdf]

Table S3. Additional analyses of the association of rs2046210 with breast cancer risk in patient subpopulations.

Category	Subcategory	dominant model OR (95% CI)	*p*	recessive model OR (95% CI)	*p*	AA vs. GG OR (95% CI)	*p*
Age *	<55	1.38 (0.95-2.45)	0.087	1.44 (0.85-2.45)	0.182	1.66 (0.94-2.94)	0.083
	≥55	0.91 (0.50-1.65)	0.748	0.84 (0-36-1.95)	0.690	0.81 (0.32-2.02)	0.554
Age **	<55	1.32 (0.92-1.90)	0.129	1.38 (0.82-2.30)	0.227	1.56 (0.90-2.71)	0.121
	≥55	1.20 (0.87-1.66)	0.264	1.09 (0.67-1.78)	0.727	1.20 (0.71-2.03)	0.493
Menopausal status	pre	1.44 (0.92-2.25)	0.111	1.47 (0.79-2.71)	0.232	1.73 (0.89-3.38)	0.115
	post	1.23 (0.90-1.69)	0.201	1.13 (0.70-1.839	0.608	1.26 (0.76-2.10)	0.377
Tumor type	ductal	1.15 (0.84-1.59)	0.374	1.27 (0.80-2.02)	0.321	1.34 (0.81-2.20)	0.255
	lobular	1.22 (0.75-1.98)	0.423	1.10 (0.53-2.27)	0.804	1.22 (0.56-2.65)	0.624
Tumor size	pT1	1.12 (0.79-1.60)	0.523	1.47 (0.89-2.44)	0.136	1.49 (0.87-2.56)	0.148
	pT2-4	1.26 (0.87-1.82)	0.225	1.05 (0.60-1.859	0.866	1.19 (0.65-2.18)	0.568
Stage	0 or I	1.11 (0.76-1.63)	0.580	1.04 (0.58-1.88)	0.889	1.10 (0.59-2.06)	0.758
	II-IV	1.25 (0.88-1.78)	0.205	1.20 (0.71-2.00)	0.503	1.33 (0.77-2.32)	0.312
Grade	pG1-2	1.26 (0.91-1.74)	0.163	1.33 (0.83-2.12)	0.235	1.47 (0.89-2.42)	0.137
	pG3	1.44 (0.97-2.12)	0.065	0.96 (0.53-1.75)	0.891	1.20 (0.63-2.28)	0.584
Lymph node status	pN0	1.29 (0.92-1.83)	0.142	1.18 (0.71-1.97)	0.518	1.35 (0.78-2.33)	0.286
	pN+	1.00 (0.67-1.48)	0.992	1.22 (0.68-2.18)	0.508	1.19 (0.64-2.20)	0.585
ER status	pos	1.24 (0.91-1.69)	0.169	1.29 (0.82-2.03)	0.268	1.42 (0.87-2.31)	0.158
	neg	1.26 (0.83-1.91)	0.269	0.96 (0.50-1.83)	0.891	1.10 (0.55-2.20)	0.787
PR status	pos	1.12 (0.79-1.59)	0.516	1.03 (0.60-1.77)	0.907	1.10 (0.62-1.94)	0.747
	neg	**1.42 (1.00-2.02)**	**0.045**	1.34 (0.81-2.19)	0.255	1.59 (0.93-2.72)	0.093
HER2 status	pos	1.31 (0.79-2.17)	0.297	1.36 (0.67-2.76)	0.405	1.53 (0.71-3.29)	0.287
	neg	1.27 (0.94-1.71)	0.125	1.17 (0.75-1.84)	0.489	1.32 (0.82-2.14)	0.259
KI67 status (%pos cells)	>10%	1.05 (0.70-1.56)	0.826	0.90 (0.47-1.73)	0.759	0.94 (0.47-1.86)	0.854
	≤10%	1.33 (0.93-1.89)	0.117	1.34 (0.81-2.22)	0.263	1.52 (0.88-2.63)	0.135
TP53 status	pos	1.30 (0.82-2.06)	0.269	1.06 (0.53-2.13)	0.874	1.22 (0.58-2.59)	0.600
	neg	1.26 (0.92-1.73)	0.148	1.29 (0.81-2.04)	0.284	1.43 (0.87-2.34)	0.159
Molecular subtype	luminal	1.17 (0.86-1.58)	0.314	1.22 (0.78-1.91)	0.391	1.30 (0.81-2.09)	0.285
	HER2 type	1.39 (0.67-2.90)	0.372	1.39 (0.51-3.77)	0.530	1.62 (0.55-4.83)	0.398
	triple neg	1.67 (0.95-2.94)	0.070	0.99 (0.43-2.30)	0.984	1.36 (0.54-3.41)	0.516

Odds ratios in the dominant model (GA+AA vs. GG), the recessive model (AA vs. GG+GA), and homozygous comparisons (AA vs. GG) of rs2046210 in the indicated clinically and molecularly relevant subcategories of breast cancer are shown. Significant associations are highlighted in bold. OR, odds ratios; 95% CI, 95% confidence intervals; *p*, *p*-values; Numbers and fraction of patients (in %) in the indicated subcategories are provided in Table 3; pre, pre-menopausal; post, post-menopausal; ER, estrogen receptor; PR, progesterone receptor; pos, positive; and neg, negative. * cases aged <55 or ≥55 years vs. controls aged <55 or ≥55 years; ** cases aged <55 or ≥55 years vs. controls of any age (for sake of comparability with the other subcategory analyses).
