# Supplementary material for: Association of the Estrogen Receptor 1 Polymorphisms rs2046210 and rs9383590 with the Risk, Age at Onset and Prognosis of Breast Cancer
Source: Cells. 2023 Feb 4;12(4):515. doi: 10.3390/cells12040515 (PMC9953811; doi:10.3390/cells12040515)
Supplement: Supplementary file 1 [file cells-12-00515-s001.zip › Table S4.pdf]

Table S4. Additional analyses of the association of rs9383590 with breast cancer risk in patient subpopulations.

Category	Subcategory	Patients		TC vs TT		CC+TC vs TT	
		No.	%	OR (95% CI)	*p*	OR (95% CI)	*p*
Age *	<55	173	42.4%	1.59 (1.00-2.54)	0.056	**1.65 (1.04-2.61)**	**0.035**
	≥55	235	57.6%	0.69 (0.32-1.51)	0.338	0.74 (0.34-1.60)	0.447
Age **	<55	173	42.4%	1.54 (0.98-2.42)	0.065	**1.60 (1.02-2.49)**	**0.042**
	≥55	235	57.6%	0.86 (0.54-1.36)	0.505	0.90 (0.57-1.41)	0.634
Menopausal status	pre	101	28.7%	1.48 (0.85-2.57)	0.171	1.53 (0.89-2.63)	0.134
	post	251	71.3%	1.01 (0.65-1.56)	0.971	1.07 (0.70-1.64)	0.754
Tumor type	ductal	249	75.5%	1.19 (0.78-1.81)	0.433	1.28 (0.84-1.93)	0.251
	lobular	81	24.5%	1.16 (0.62-2.20)	0.643	1.15 (0.61-2.16)	0.677
Tumor size	pT1	175	52.2%	1.02 (0.63-1.67)	0.927	1.04 (0.64-1.70)	0.860
	pT2-4	160	47.8%	1.36 (0.85-2.19)	0.211	1.43 (0.89-2.27)	0.142
Stage	0 or I	143	43.2%	0.76 (0.43-1.34)	0.334	0.79 (0.45-1.38)	0.402
	II-IV	188	56.8%	1.34 (0.85-2.10)	0.212	1.39 (0.89-2.17)	0.151
Grade	pG1-2	239	62.1%	1.09 (0.70-1.68)	0.710	1.07 (0.69-1.65)	0.762
	pG3	146	37.9%	1.30 (0.79-2.13)	0.311	1.42 (0.88-2.30)	0.161
Lymph node status	pN0	196	59.6%	0.90 (0.55-1.47)	0.673	0.95 (0.59-1.53)	0.840
	pN+	133	40.4%	1.38 (0.83-2.29)	0.220	1.46 (0.89-2.40)	0.138
ER status	pos	273	69.6%	1.16 (0.77-1.76)	0.473	1.17 (0.78-1.76)	0.454
	neg	119	30.4%	1.08 (0.62-1.89)	0.788	1.18 (0.68-2.02)	0.562
PR status	pos	187	48.6%	1.11 (0.69-1.77)	0.672	1.09 (0.68-1.74)	0.720
	neg	198	51.4%	1.13 (0.72-1.80)	0.592	1.22 (0.78-1.91)	0.391
HER2 status	pos	74	19.9%	1.19 (0.62-2.29)	0.612	1.17 (0.61-2.25)	0.645
	neg	297	80.1%	1.09 (0.72-1.64)	0.690	1.14 (0.76-1.70)	0.532
KI67 status (%pos cells)	>10%	125	40.6%	1.02 (0.58-1.78)	0.951	1.11 (0.65-1.90)	0.712
	≤10%	183	59.4%	1.06 (0.65-1.70)	0.824	1.08 (0.67-1.73)	0.764
TP53 status	pos	98	25.7%	1.47 (0.84-2.57)	0.191	1.59 (0.92-2.74)	0.104
	neg	284	74.3%	1.05 (0.69-1.60)	0.807	1.06 (0.70-1.60)	0.782
Molecular subtype	luminal	295	82.6%	1.17 (0.78-1.75)	0.449	1.17 (0.79-1.75)	0.435
	HER2 type	33	9.2%	1.50 (0.62-3.60)	0.380	1.48 (0.61-3.55)	0.398
	triple neg	62	17.4%	0.86 (0.39-1.89)	0.699	1.05 (0.51-2.18)	0.886

Odds ratios in TC vs. TT comparisons and in the dominant model (CC+TC vs. TT) for rs9383590 in the indicated clinically and molecularly relevant subcategories of breast cancer are shown. Significant associations are highlighted in bold. OR, odds ratios; 95% CI, 95% confidence intervals; *p*, *p*-values; No., number of patients in the indicated subcategories; %, fraction of patients in the indicated subcategories (in %); pre, pre-menopausal; post, post-menopausal; ER, estrogen receptor; PR, progesterone receptor; pos, positive; and neg, negative. * cases aged <55 or ≥55 years vs. controls aged <55 or ≥55 years; ** cases aged <55 or ≥55 years vs. controls of any age (for sake of comparability with the other subcategory analyses).
