# Supplementary material for: Association of the Estrogen Receptor 1 Polymorphisms rs2046210 and rs9383590 with the Risk, Age at Onset and Prognosis of Breast Cancer
Source: Cells. 2023 Feb 4;12(4):515. doi: 10.3390/cells12040515 (PMC9953811; doi:10.3390/cells12040515)
Supplement: Supplementary file 1 [file cells-12-00515-s001.zip › Table S5.pdf]

Table S5. Age-adjusted analyses of the association of rs9383590 with breast cancer risk in patient subpopulations.

Category	Subcategory	TC vs TT OR (95% CI)	TC vs TT p	CC+TC vs TT OR (95% CI)	CC+TC vs TT p	C vs T OR (95% CI)	C vs T p
Age *	<55	1.61 (0.95-2.71)	0.076	1.64 (0.98-2.75)	0.060	1.62 (0.99-2.64)	0.054
	≥55	0.65 (0.29-1.44)	0.297	0.69 (0.31-1.51)	0.361	0.75 (0.36-1.59)	0.466
Age **	<55	1.53 (0.97-2.41)	0.073	**1.58 (1.01-2.48)**	**0.048**	**1.60 (1.04-2.45)**	**0.034**
	≥55	0.60 (0.28-1.30)	0.196	0.63 (0.30-1.36)	0.243	0.69 (0.34-1.43)	0.322
Menopausal status	pre	1.48 (0.85-2.57)	0.172	1.53 (0.89-2.63)	0.135	1.54 (0.92-2.59)	0.109
	post	0.98 (0.50-1.93)	0.949	1.08 (0.56-2.10)	0.810	1.20 (0.64-2.22)	0.574
Tumor type	ductal	1.19 (0.72-1.96)	0.496	1.27 (0.78-2.07)	0.332	1.33 (0.85-2.10)	0.216
	lobular	1.16 (0.54-2.51)	0.705	1.15 (0.53-2.49)	0.719	1.14 (0.53-2.44)	0.737
Tumor size	pT1	1.11 (0.63-1.96)	0.718	1.13 (0.64-1.98)	0.671	1.14 (0.67-1.96)	0.629
	pT2-4	1.27 (0.71-2.24)	0.423	1.37 (0.79-2.40)	0.269	1.45 (0.87-2.45)	0.162
Stage	0 or I	0.79 (0.41-1.52)	0.472	0.82 (0.43-1.56)	0.542	0.86 (0.47-1.59)	0.637
	II-IV	1.34 (0.78-2.30)	0.294	1.43 (0.84-2.43)	0.190	1.49 (0.91-2.45)	0.120
Grade	pG1-2	1.22 (0.71-2.10)	0.467	1.21 (0.71-2.07)	0.491	1.19 (0.70-2.029	0.524
	pG3	1.29 (0.74-2.25)	0.376	1.42 (0.83-2.44)	0.202	1.52 (0.93-2.48)	0.102
Lymph node status	pN0	1.00 (0.57-1.76)	0.995	1.07 (0.61-1.85)	0.820	1.13 (0.67-1.89)	0.648
	pN+	1.26 (0.70-2.27)	0.453	1.32 (0.74-2.36)	0.353	1.37 (0.79-2.37)	0.270
ER status	pos	1.38 (0.82-2.34)	0.229	1.39 (0.82-2.34)	0.219	1.37 (0.83-2.28)	0.220
	neg	1.07 (0.59-1.94)	0.819	1.18 (0.66-2.09)	0.578	1.28 (0.75-2.17)	0.372
PR status	pos	1.12 (0.63-2.01)	0.698	1.11 (0.62-1.99)	0.724	1.10 (0.62-1.95)	0.756
	neg	1.23 (0.73-2.08)	0.447	1.33 (0.80-2.22)	0.278	1.40 (0.87-2.25)	0.166
HER2 status	pos	1.33 (0.66-2.68)	0.427	1.32 (0.66-2.64)	0.445	1.29 (0.65-2.56)	0.473
	neg	1.09 (0.66-1.80)	0.738	1.16 (0.71-1.90)	0.548	1.22 (0.77-1.94)	0.388
KI67 status (%pos cells)	>10%	1.07 (0.58-2.00)	0.823	1.18 (0.64-2.15)	0.601	1.27 (0.73-2.21)	0.410
	≤10%	1.11 (0.63-1.98)	0.718	1.16 (0.65-2.04)	0.620	1.19 (0.69-2.05)	0.525
TP53 status	pos	1.32 (0.72-2.42)	0.372	1.46 (0.81-2.62)	0.216	1.56 (0.91-2.68)	0.115
	neg	1.24 (0.74-2.09)	0.409	1.25 (0.75-2.08)	0.397	1.24 (0.76-2.04)	0.395
Molecular subtype	luminal	1.33 (0.81-2.21)	0.266	1.33 (0.81-2.20)	0.260	1.32 (0.81-2.15)	0.263
	HER2 type	1.63 (0.65-4.07)	0.314	1.61 (0.64-4.03)	0.324	1.57 (0.64-3.86)	0.343
	triple neg	0.84 (0.37-1.89)	0.662	1.04 (0.49-2.21)	0.915	1.26 (0.65-2.46)	0.505

Age-adjusted odds ratios in TC vs. TT comparisons, in the dominant model (TC+CC vs. TT), and per-allele odds ratios (C vs. T) for rs9383590 in the indicated clinically and molecularly relevant subcategories of breast cancer are shown. Significant associations are highlighted in bold. OR, odds ratios; 95% CI, 95% confidence intervals; *p*, *p*-values; Numbers and fraction of patients (in %) in the indicated subcategories are provided in Table S4; pre, pre-menopausal; post, post-menopausal; ER, estrogen receptor; PR, progesterone receptor; pos, positive; and neg, negative. * cases aged <55 or ≥55 years vs. controls aged <55 or ≥55 years; ** cases aged <55 or ≥55 years vs. controls of any age (for sake of comparability with the other subcategory analyses).
