# Supplementary material for: Association of the Estrogen Receptor 1 Polymorphisms rs2046210 and rs9383590 with the Risk, Age at Onset and Prognosis of Breast Cancer
Source: Cells. 2023 Feb 4;12(4):515. doi: 10.3390/cells12040515 (PMC9953811; doi:10.3390/cells12040515)
Supplement: Supplementary file 1 [file cells-12-00515-s001.zip › Table S6.pdf]

Table S6. Univariable analyses of the overall, disease-free and metastasis-free survival using a Cox proportional hazards model.

		rs9383590		rs2046210	
**Survival type**	**Patients**	**HR (95% CI)**	***p***	**HR (95% CI)**	***p***
Overall survival	all	0.94 (0.54-1.61)	0,810	0.91 (0.65-1.28)	0.593
	ER pos	1.01 (0.47-2.19)	0.978	0.83 (0.54-1.29)	0.418
	ER neg	0.78 (0.36-1.69)	0.536	0.98 (0.56-1.72)	0.943
	luminal A	0.67 (0.26-1.75)	0.417	0.74 (0.44-1.23)	0.246
Disease-free survival	all	0.90 (0.52-1.58)	0.724	0.92 (0.65-1.29)	0.621
	ER pos	0.73 (0.31-1.75)	0.487	0.82 (0.53-1.29)	0.399
	ER neg	0.90 (0.43-1.86)	0.771	1.00 (0.57-1.76)	0.990
	luminal A	0.41 (0.12-1.34)	0.140	0.66 (0.38-1.16)	0.146
Metastasis-free survival	all	0.93 (0.51-1.69)	0.818	0.87 (0.60-1.27)	0.469
	ER pos	0.70 (0.27-1.82)	0.469	0.72 (0.44-1.20)	0.210
	ER neg	1.03 (0.48-2.22)	0.931	1.09 (0.60-2.00)	0.770
	luminal A	0.32 (0.08-1.35)	0.121	0.50 (0.26-0.97)	0.037

Analyses of the overall survival, disease-free survival and metastasis-free survival were done in all patients, ER pos patients, ER neg patients, and luminal A patients as indicated. Genotypes were coded as follows: rs9383590, TT=0 (common homozygote genotype), TC=1, CC=2; rs2046210, GG=0 (common homozygote genotype), GA=1, AA=2. HR, hazard ratios; 95% CI, 95% confidence intervals; *p*, *p*-values; ER, estrogen receptor; pos, positive; neg, negative.
